# The Prevalence and Correlates of Anxiety, Stress, Mood Disorders, and Sleep Disturbances in Poland after the Outbreak of the Russian–Ukrainian War 2022

**DOI:** 10.3390/healthcare12181848

**Published:** 2024-09-14

**Authors:** Karolina Hoffmann, Michał Michalak, Dorota Kopciuch, Wiesław Bryl, Krzysztof Kus, Elżbieta Nowakowska, Anna Paczkowska

**Affiliations:** 1 Department of Internal Diseases, Metabolic Disorders and Arterial Hypertension, Poznan University of Medical Sciences, 61-701 Poznań, Poland; 2 Department of Computer Science and Statistics, Poznan University of Medical Sciences, 60-806 Poznań, Poland; 3 Department of Pharmacoeconomics and Social Pharmacy, Poznan University of Medical Sciences, 60-806 Poznań, Poland; 4 Department of Pharmacology and Toxicology Institute of Health Sciences, Collegium Medicum, University of Zielona Góra, 65-417 Zielona Góra, Poland

**Keywords:** military conflict, Russian–Ukrainian War, mental health, anxiety, depression, sleep disorders

## Abstract

Background: The conflict of the Russian–Ukrainian War that began on 24 February 2022 has profoundly changed Europe. The primary objective of this study was to assess the prevalence of anxiety, stress, depression, and insomnia among a group of surveyed Poles in the first months after the outbreak of war in 2022. The secondary goal was to analyze potential risk factors for these mental disorders. Methods: A cross-sectional survey-based study was conducted. An anonymous questionnaire was created using Google Forms and distributed through social media from March 2022 to June 2022. The questionnaire included the Depression, Anxiety and Stress Scale (DASS-21) and an evaluation of the Insomnia Severity Index (ISI). Results: Overall, 11.26% of 311 participants had depression, 10.29% had anxiety, and 24.12% experienced stress. Further, 62.05% of them declared sleep disturbances, and about 60% of them reported experiencing fears associated with the war. The outcomes of the assessment of psychiatric symptoms (depression, anxiety, stress and sleep disturbance) were associated with following factors: self-reported health status, fear of Russian invasion of Ukraine, and fear of the war extending to Poland. When the results for psychiatric symptoms were categorized into two groups, severe and non-severe, logistic regression analysis was only feasible for the insomnia variable. For this variable, multivariate logistic regression identified key potential factors: age, stress, and fear of Russian invasion of Ukraine. Conclusions: The respondents were found to be highly concerned about the war. In total, almost half of them manifested symptoms of anxiety, depression, and/or stress. Slightly less than two-thirds had sleep disorders. This study confirms that in a crisis situation, mental health screening is necessary.

## 1. Introduction

More than two years after the World Health Organization (WHO) declared the COVID-19 pandemic, the world faced another humanitarian threat: an armed conflict between the Russian Federation and the country of Ukraine. This era of the Russian–Ukrainian War (RUW), which began on 24 February 2022, has profoundly changed Europe. Every armed conflict is responsible for unpredictable, in terms of scale, traumatic experiences within societies directly or indirectly affected by war [1]. Not only are the lives of adults involved in the armed conflict at stake, but also the lives of children, youth, and the next generation, which is why war is always “a public health emergency that spans many years” [2]. In early March 2022, it became clear that “this war is not just about Ukraine and Russia”, as it has a profound impact on global security and sustainable growth [3]. Therefore, the RUW is seen as a 21st century man-made disaster, defined by the WHO as a “[…] phenomenon of sufficient magnitude to require external assistance” [4]. Although its serious psychological consequences for the Ukrainian people and European citizens are now being documented and studied, they will no doubt be further explored in the unknown future, when the RUW goes down in history [5].

The RUW has turned out to be a test of the world’s solidarity with Ukraine, as well as a challenge for neighboring countries, who, without delay or doubt, showed the true meaning of the proverb “A friend in need is a true friend”. According to data gathered by the United Nations High Commissioner for Refugees (UNHCR, the UN Refugee Agency), up until now, 8.1 million Ukrainian refugees have been recorded across Europe [6]. Since the first days of the conflict, refugees from Ukraine have found great support from many European governments and institutions, and ordinary people have opened their homes and hearts to victims of the Russian invasion. On 3 March 2022, the Ministry of the Interior and Administration of the Republic of Poland presented a bill that was immediately adopted by the Parliament (the Act on Assistance to Ukrainian Citizens in Connection with the Armed Conflict in Ukraine) [7]. Poland quickly organized all kinds of help for Ukrainian refugees. In the period of 24 February 2022 to 30 June 2022, 70.2% of households in Poland provided assistance to residents of Ukraine [6]. At the end of May 2022, Poland ranked first in terms of the number of Ukrainian refugees it had hosted (3.5 million) [6]. By 7 March 2023, about 1.5 million refugees had registered with the Polish authorities, receiving a state Polish ID number (PESEL) [6]. As of 19 August 2024, 6,739,400 refugees from Ukraine have been recorded globally, including 6,168,100 in Europe and 957,505 in Poland [6].

Ukrainian citizens, being on the first front line, are most affected by the RUW, but the impact of this armed conflict on neighboring countries, including Poland, should be explored [8]. A few months after the 2022 outbreak of the RUW, more than two-thirds of surveyed Poles admitted to fear of the future [9]. It was proven that both victims and helpers of victims are at a risk of developing posttraumatic stress disorder (PTSD) [10,11]. Potential behavioral and psychological symptoms after a disaster include anxiety, depression, various stress responses, and emotional instability [8,12].

The latest large-scale epidemiological studies on the prevalence of depression and anxiety disorders among the Polish population reveal that this health issue affects a significant majority of the surveyed population. Compared to previous Polish studies and other diverse cultural samples, the prevalence of probable anxiety and depression disorders in Polish adults in July 2023 was extremely high: in the entire sample, 59.28% and 52.91% of the respondents screened positively for anxiety and depression, respectively [13].

Charlson et al. reported that people affected by war are more likely to suffer from mental problems [14]. Every 11th person (9.1%) who has experienced an armed conflict in the last decade suffers from moderate or severe mental disorders [14]. Moreover, the effects of war are spread over time and space, and can be serious even for people living far from the front lines of the armed conflict, reaching far beyond the areas directly affected by war [15,16]. This observation can be explained by, among other factors, the heightened exposure of individuals to violence and suffering due to reports of armed conflicts and images presented on social media. Since February 2022, millions of followers have been collecting information about the RUW on social media [17]. It has been reported that citizens of countries neighboring Russia or Ukraine, and even those living far from these areas, are at risk of experiencing a deterioration of their mental health as a consequence of the RUW [16,17]. Moshagen et al. examined psychological responses to the RUW in Poland, Germany, the United Kingdom, and the United States of America (USA), finding that residents of each of these countries reported feelings of anger, threat, and anxiety related to the RUW outbreak [18].

It is estimated that approximately 191 million people died as a result of conflicts in the 20th century, which is almost half of Europe’s current population. The conflicts in Rwanda and Kosovo in the 1990s alone accounted for as much as 90 percent of civilian deaths [19]. At least 117.3 million people around the world have been forced to flee their homes. Among these people, almost 43.4 million are refugees, of which about 40 percent are under 18 years old [20]. Initial estimates suggest that for every person killed directly by war, nine people will be killed indirectly, although much will depend on the nature of the conflict and underlying health conditions in the countries where the war is taking place [21]. Since February 2022, Ukraine has not been protected from attacks on its healthcare facilities, and hence the RUW’s impact on Ukrainian healthcare and public health has also been significant. In a war zone, the flow of essential health goods is interrupted and medical staff and patients are unable to move around, meaning that health outcomes can deteriorate rapidly [15]. Numerous recent or ongoing armed conflicts and wars (e.g., in Afghanistan, Colombia, Iraq, Somalia, Sudan, and Syria) have left a large number of affected people unable to escape memories of the horrors from which they fled, namely threats, violence, and witnessing the murder of loved ones [22]. In addition to those directly affected by war, vast numbers of civilians may also be deeply affected indirectly. In the age of digital connectivity, people can experience and learn about war through news and social media, as well as from any refugee friends and family members they have, which are no doubt growing in number. Lai et al. estimated multiple distress symptoms in families exposed to the Gulf Crisis (1990–1991), and showed that parent distress was a risk factor for depression in their children [23].

The impact of war on neighboring countries and even distant nations can also be economic. The effects of the war in Ukraine pose significant risks to World Bank Group (WBG)’s poorest and most vulnerable clients, exacerbating the effects of simultaneous crises—in particular, the pandemic and climate change [24]. The immediate impact of the RUW on food security was felt by countries largely dependent on imports from Ukraine and Russia, and by importers more broadly, given the sharp increase in global wheat prices [24]. Global hunger affected around 9.2% of the world population in 2022, compared with 7.9% in 2019 [25]. In 2022, about 30% of the global population (2.4 billion people) were moderately or severely food-insecure. In 2030, almost 600 million people will suffer from hunger—23 million more than in a scenario without the RUW [25].

From the other point of view, thanks to the influx of people from Ukraine, many industrial sectors have solved their labor shortage problems [26]. Refugees from Ukraine that came to Poland started working quickly. The estimated employment rate among refugees from Ukraine in Poland 5 months after the 2022 outbreak of the RUW was high, amounting to 30% [26]. At the end of June 2024, over 771 thousand Ukrainians were working in Poland. For comparison, in March 2022, the number of insured citizens of this country was 666.7 thousand, 39.7 thousand more than in December 2021, before the RUW [27]. Most foreigners working in Poland come from Ukraine, with Belarusians coming in second place (134 thousand) and Georgians in third place (27.2 thousand).

Ukrainian refugees’ integration into the European labor market is estimated as successful, as their employment rate ranges from more than 10% to well above 40% [28,29]. Their employment rate is particularly high in Poland, at more than 60% currently [28,30]. Important barriers to their employment, which have already been identified, include lack of knowledge of the host country’s language, lengthy recognition procedures and potential job mismatches, lack of employment opportunities/information, and lack of childcare [28]. The language barrier appears to be a clearly visible and key obstacle in the labor markets of all countries [29]. In each country hosting Ukrainian people, the qualification recognition process has been accelerated, but, even with this, refugees must wait about two years to find employment. The costs of the employment procedure also contribute to the challenges [28]. Structural mismatches lead to refugees undertaking work for which they are overqualified: despite their generally high qualification levels, 50% perform simple jobs [26]. Lack of knowledge about job opportunities and other related information may also reduce refugees’ employability [31]. Finally, care responsibilities are particularly important in the case of Ukrainian refugees, considering their composition—that is, considering the predominance of women, who are often accompanied by children, and elderly people who also need care [26]. In many countries, including Poland, opportunities for flexible work schedules are limited, which worsens their labor market situation [32].

Rizzi et al. analyzed data from 13 studies, giving a comprehensive spectrum of factors influencing the resilience of Ukrainian refugees [33]. Among them, mental disorders, financial instability, experiences of loved ones’ distress or the displacement of loved ones, and acclimatization to unfamiliar environments have emerged as key determinants affecting the risk and severity of mental health problems. The protective role of coping strategies, social connections, faith-based mechanisms, self-efficacy, and cultural resilience has been demonstrated [33]. What is more, maintaining communication with separated loved ones, as well as experiencing the company of helpers and hosts, has emerged as a key element of coping and resilience [34].

In a study conducted by Pavlova et al., a significant proportion of Ukrainian students showed moderate and high levels of resilience (46.0% and 14.5%, respectively). Among other factors, the interplay of optimism, hope, resilience, and the use of emotional support emerged as a robust predictor of life satisfaction in the study population [35]. In the period spanning September to October 2022, a cohort of Ukrainian civilians participated in another survey, estimating, among other things, symptoms of peritraumatic experiences, depression, perceived social support, and resilience [36], wherein Palace et al. proved the presence of a “war stress sharing deterioration effect”. They showed that perceived social support, if it comes from people facing the same terrible reality, can increase the peritraumatic distress experienced by individuals. Essentially, the study uncovered a paradoxical dimension in which support intended to alleviate psychological distress may itself unintentionally contribute to the escalation of stressors, highlighting the complexity of social dynamics in the context of conflict [36].

Therefore, the study presented here was aimed at evaluating selected indicators of the mental state of surveyed Polish citizens during the first months following the 2022 outbreak of the RUW. Our primary objective was to assess the prevalence of anxiety, stress, mood disorders, and sleep disturbances in the study population; the secondary objective was to analyze the potential risk factors for mental disorders among the surveyed Poles.

## 2. Materials and Methods

### 2.1. Study Design and Population

A cross-sectional survey-based study was conducted. Participants were selected based on the following inclusion criteria: being of legal age, residing in Poland, providing voluntary and informed consent to participate, and having internet access. The exclusion criteria were as follows: being under 18 years old, residing outside of Poland, refusing to provide informed consent, and lacking internet access.

### 2.2. Tools

Due to the restrictions related to COVID-19 in force in Poland during the pandemic, as well as in order to minimize the risk of SARS-CoV-2 infection among the study participants, the survey was conducted using the Computer-Assisted Web Interview (CAWI) method, acceptable as a research tool [37,38]. An anonymous questionnaire was created using Google Forms and distributed from March 2022 to June 2022. Potential respondents were able to download the link to the study questionnaire from the Poznan University of Medical Sciences website promoting the study project and from social media (Facebook, LinkedIn). The study tool consisted of mandatory fields, ensuring that no answers were omitted. To prevent multiple submissions being submitted by the same participant, the Google Forms platform recognized the IP address of the interviewer’s device. The questionnaire included the Depression, Anxiety and Stress Scale (DASS-21) and an Insomnia Severity Index (ISI) evaluation [39,40,41]. The final results regarding the assessment of depression, anxiety, and stress were calculated by summing the scores for the relevant questions and then multiplying the total by 2. The overall score for the levels of depression, anxiety, and stress was interpreted according to the standardized scoring key for the DASS-21 questionnaire [39]. Similarly, the interpretation of the severity of sleep disorders was based on the standardized scoring key for the Insomnia Severity Index [40]. The study tool is available in the Supplementary Materials (S1: “Study questionnaire”).

### 2.3. Ethics

The authors assert that all procedures contributing to this work complied with the ethical standards of the relevant national and institutional committees on human experimentation and with the Helsinki Declaration of 1975, as revised in 2008 [42]. All procedures involving human subjects/patients were approved on 17 March 2022 by the Bioethics Committee of Poznan University of Medical Sciences (Poznań, Poland). The Bioethics Committee issued a statement affirming that this study had no features of a medical experiment. Prior to their enrollment, volunteers were instructed about the study objectives. Participants were able to withdraw their consent at any time and leave the study. Because no personal data of the respondents were collected in order to keep their identities anonymous, this research project does not violate the Personal Data Protection Act.

### 2.4. Statistical Analyses

We performed comparisons for the depression, anxiety, stress, and insomnia indices based on the analyzed categorical factors. The Mann–Whitney test was used when the dependent variable was binary, while the Kruskal–Wallis test was employed for variables with more than two categories. For significant results, post hoc analysis was conducted using the Dunn test. These results were recorded as median values (Me) and interquartile ranges (Q_1_–Q_3_).

Additionally, we categorized the results for depression, anxiety, stress, and insomnia into two groups: severe and non-severe. However, logistic regression analysis was only feasible for the insomnia variable. For this variable, both univariate and multivariable logistic regression analyses were performed to identify potential factors influencing insomnia. The multivariable logistic regression model, which allows for the analysis of several predictors simultaneously while controlling for potential confounding variables, was assessed using a stepwise backward selection procedure. Each result from the logistic regression analyses was recorded as an odds ratio (OR) with a 95% confidence interval (95% CI).

All statistical analyses were conducted using Statistica data analysis software v. 13.3 (TIBCO Software Inc., 2017, Palo Alto, CA, USA), “http://statistica.io (assessed on 31 July 2022)”. A *p*-value of less than 0.05 was considered statistically significant for all tests.

## 3. Results

There were 311 respondents in the study group, including 52.09% women. The average age of the surveyed Poles was 33.45 ± (13.53). Just under two-thirds had received higher education, and more than half were professionally active. The details of the characteristics of the respondents are presented in Table 1.

Overall, 11.26% of participants had a mild, moderate, or severe level of depression, whereas 10.29% had a mild or moderate level of anxiety. Further, 24.12% of the surveyed Polish citizens experienced a mild or moderate level of stress. The majority of participants (62.05%) declared that they experienced sleep disturbances. The specific results are presented in Table 2.

More than 60% of participants experienced fears associated with the outbreak of the armed conflict in Ukraine. Detail data on this are provided in Table 3.

We performed comparisons for the depression, anxiety, stress, and insomnia indices based on the analyzed categorical factors. Significant results were obtained for comparisons between the following: education and level of anxiety; self-reported health status and all four mental disorders; fear of the armed conflict in Ukraine and anxiety, stress, and insomnia; fear of the armed conflict extending to Poland and depression and stress. For those significant results, post hoc analysis was conducted. This analysis did not reveal any homogenous groups when analyzing education and fear of the armed conflict in Ukraine as two factors regarding anxiety levels. The severity of depression was influenced by the self-reported health status (*p* < 0.001) and fear of the armed conflict extending to Poland (*p* = 0.024). In the post hoc analysis, the only variable differentiating the level of anxiety was the self-reported health status (*p* < 0.001). In relation to the level of stress, three factors were significant: self-estimated health status (*p* < 0.001), fear of the armed conflict in Ukraine (*p* < 0.001), and fear of the armed conflict extending to Poland (*p* < 0.001). In the case of insomnia, the severity of symptoms was influenced by two variables: self-reported health status (*p* < 0.001) and fear of the armed conflict in Ukraine (*p* = 0.003). Data on the psychiatric symptoms are presented in Table 4.

The results for depression, anxiety, stress, and insomnia were categorized into two groups: severe and non-severe. Logistic regression analysis was only feasible for the insomnia variable. For this variable, both univariate and multivariable logistic regression analyses were performed in order to identify potential factors influencing insomnia.

In the analysis of the univariate logistic regression model, factors such as age, self-reported health status, depression, anxiety, and stress indicated the risk of insomnia in the analyzed group. However, in the case of the multivariate model, three factors remained significant: age (OR: 1.04 [95% CI: 1.01, 1.07]), fear of the armed conflict in Ukraine (OR: 2.85 [95% CI: 1.02, 7.96]), and stress (OR: 1.40, [1.24, 1.59]). This suggests that after taking into account the coexistence of the analyzed factors, these are the strongest predictors of insomnia. The logistic regression data are presented in Table 5.

## 4. Discussion

### 4.1. The Prevalence of Depression, Anxiety, and Stress

The current study found that approximately one in ten study participants had mild, moderate, or severe levels of depression, and a similar proportion of the study group had mild or moderate levels of anxiety. About a quarter of the surveyed Poles experienced mild or moderate levels of stress. Our result for the percentage of people with mental health disorders is lower than that found in another study in a German population examined in the months following the outbreak of RUW [43]. In this German study, comparing the results from the first weeks of the RUW with the results obtained 6 months later, a decrease in the frequency of anxiety was observed (from 27.3% to 16%), while the percentage of declared depressive symptoms changed insignificantly (from 19.5% to 16.4%) [43]. However, Massag et al. used different study tools: the Generalized Anxiety Disorder Assessment (GAD-7) for anxiety and the modified Peritraumatic Distress Inventory (PDI) for distress. Another study conducted among Czech students showed that after the outbreak of the RUW, 34% and 40.7% of participants, respectively, had moderate to severe levels of anxiety and depression [5]. This is a higher prevalence than that found in our study, but it should be noted that these authors also used different research tools: GAD-7 for anxiety and the Patient Health Questionnaire (PHQ-9) for depression [5].

Previously, it has been observed that the proximity of a country to a region engulfed in armed conflict leads to a greater awareness of war in that country, which is thus a source of more frequent occurrences of mental disorders among its citizens. For example, in research comparing the Czech Republic and Slovakia, the latter country was found to have a higher amount of people “feeling concerned by the RUW”, which was explained by the larger influx of refugees in Slovakia [5,6]. Our study in Poland found a lower rate of mental disorders following the RUW, and so further multi-country research is needed to determine whether geographical proximity plays a role in modifying the level of perceived risk.

Psychological reactions to the RUW were also explored by Moshagen et al. using population sample sizes similar to that used in our study (between 291 and 297 participants by country) [18]. These researchers concluded that among all individuals, they found emotional involvement, willingness to host Ukrainian people, and an agreement to sanctions against the Russian Federation. This study also confirmed that the surveyed Polish citizens supported the imposition of sanctions on the Russian Federation, which can be partly explained by the history of mutual relations between Poland and Russia and Poland’s history of being the country to organize help for the largest group of Ukrainian refugees. Participants from Poland reported the highest level of perceived threat and a level of anxiety similar to that of the surveyed Germans [18]. In this study, anger (e.g., “I feel angry”) and perceived threat (e.g., “I feel safe”) were assessed with two items each. Anxiety (e.g., “I feel worried”) and empathy (e.g., “I try to imagine what people in Ukraine are going through”) were measured using three items each. All items were answered on a five-point scale ranging from “strongly disagree” to “strongly agree”.

Limone et al. performed a meta-analysis to assess the impact both of the COVID-19 pandemic (30 included studies) and the RUW (2 included studies) on stress and anxiety in post-secondary students [44]. They found that the prevalence of anxiety was relatively high, ranging from 88.9 to 13.63%, and the prevalence of stress ranged from 56 to 28.14%. A majority of the analyzed studies reported anxiety prevalence rates above 50% and stress prevalence between 20 and 40% [44]. These results also show a higher prevalence than that observed in our study, which included not only students but people from various age groups, with the mean age being 33.45 ± 13.53. Other instruments used to measure anxiety and stress after the outbreak of the RUW have included the Patient Health Questionnaire 4 (PHQ-4), used alongside GAD-7 in a study conducted by Riad et al. [5], and the State-Trait Anxiety Inventory, used in a study performed by Skwirczyńska et al. in 2023 [45].

A similar proportion of people affected by these mental health conditions was shown in a meta-analysis by Lim et al., who found that the combined prevalence of depression, anxiety, and posttraumatic stress in populations experiencing war was 28.9, 30.7, and 23.5%, respectively [46]. Compared to the military, civilians were significantly more likely to suffer from anxiety and depression, but not posttraumatic stress. The most common depression assessment tools used were PHQ-8 and PHQ-9 [46]. An even higher proportion of civilians with severe depression was found (47.1%) among Palestinian adults living in the Gaza Strip [47]. Msaad et al. used the following study tools: the World Health Organization’s Five Well-Being Index (WHO-5) and PHQ-9. 

Compared to the present study, a slightly higher percentage of mental health issues after armed conflicts was found by Barzoki et al. [48]. In their meta-analysis, the authors included data from studies varying in the measurement tools they used, including, e.g., the Short Post-Traumatic Stress Disorder Rating Interview (SPRINT), the SPAN Self-Report Screen (a four-item self-report screening test derived from the Davidson Trauma Scale), the Trauma Screening Questionnaire (TSQ), and the Primary Care PTSD Screen for DSM-5 (PC-PTSD-5). However, the associations between the type of measurement tool used and the obtained results were not explored [48].

A slightly lower prevalence of mental disorders than in the present study was found in conflict areas in Nepal: about 9% of 720 adults met the threshold for PTSD, 27.5% for depression, and 22.9% for anxiety [49]. The outcome measures used in this study in Nepal were locally validated with the Beck Anxiety Inventory (BAI), the Beck Depression Inventory (BDI), the Post-Traumatic Stress Disorder (PTSD) Checklist—Civilian Version (PCL-C), and a locally constructed function impairment scale assessing resources and coping strategies.

Currently, it is underlined that a holistic approach covering all factors (cultural, socioeconomic, political, physiological, psychological, and spiritual) is the only way to scale up mental health services and facilitate their better utilization [50]. For example, in a study conducted in Northern India on patients with mental health disorders, it was shown that the inclusion of psychosocial interventions in pharmacological treatments increased their overall efficacy [51]. Cultural and social factors have the most direct role in the causation of major depression and posttraumatic stress disorder [52]. Some studies reveal alarming rates of PTSD in communities with a high degree of pre-immigration exposure to trauma [52,53]. For example, in some samples, up to 70 percent of refugees from Vietnam, Cambodia, and Laos have been found to meet the diagnostic criteria for PTSD [52].

Some studies have indicated a two- to four-fold elevated risk of psychotic disorders among some migrant groups, relative to the non-migrant host population, with variation by host country and country of origin [54,55]. One Canadian study showed that the prevalence of mood or anxiety disorders among migrants increases with more time spent in Canada, but this was found to vary greatly by region of birth and migrant class [55]. Dom et al. stressed that after the 2008 economic crisis, mental health problems became more common due to risk factors such as job loss and long-term unemployment, as well as pre-existing vulnerabilities [56]. Interesting findings were presented by Kasinger et al., who explored historical and regional particularities in the prevalence of traumatic events and posttraumatic stress disorder in East and West Germany. Significant differences in the prevalence of PTSD were only observed between different age cohorts. In this study, Kasinger et al. found that older participants who had experienced World War II had a higher risk of suffering from PTSD.

In a Latin American country with a military dictatorship where torture is systematically used against regime dissidents, the profile of trauma survivors will be different from that of survivors of genocide in Africa where civilians have generally been victims of murder and human rights violations. Trauma appears to be cumulative, although the type of trauma can also impact mental health [57].

### 4.2. The Prevalence of Sleep Disorders

In this study, the majority of participants (62.05%) reported sleep disorders. In a systematic review by Richter et al., it was found that the prevalence of sleep disorders in conflict-affected areas, as measured among migrants and refugees, ranged from 39 to 99% [58]. Msaad et al. used different tools than those used our study (the Pittsburgh Sleep Quality Index (PSQI) and the Epworth Sleepiness Scale (ESS)) to examine sleep disturbances in February–March 2022 among Palestinian civilians living in the Gaza Strip [47]. Poor sleep quality was found in half of the respondents (52.8%), defined as PSQI ≥ 6, and in almost one-third of them (30.5%), defined as PSQI ≥ 8. It was shown that 43.6% and 26.4% of respondents complained of excessive daytime sleepiness (EDS) and short sleep duration, respectively [47].

Lavie et al. analyzed data collected from civilians who experienced Scud missile attacks during the Gulf War. They used a study questionnaire including three sleep-related questions. Overall, 28% of participants had a sleep disorder: 10% awakened during sleep, 4.5% had difficulty falling asleep, and 13.5% had a combination of the two [59]. A relatively low rate (1%) of sleep disorders was detected in a population of 536 civilians exposed to constant rocket attacks during the Second Lebanon War [60]. However, the majority of participants (90%) complained of anxiety.

### 4.3. Variables Influencing Psychiatric Symptoms (Depression, Anxiety, Stress, and Sleep Disturbance)

In the present study, the following variables significantly influenced the respondents’ mental state: self-estimated health condition, fear of the RUW, and fear of this war extending to Poland. Respondents who reported a “very good” health status had lower levels of depression, anxiety, stress, and insomnia when compared to people describing their health status as “good” or “not very good”. Similar results were obtained when comparing between the “good” and “not very good” self-assessments of health. People who declared that they did not feel fear of the armed conflict in Ukraine were characterized by lower levels of stress and insomnia. Respondents who did not feel fear of the armed conflict extending to Poland had lower levels of depression and stress.

The worse the participants’ declared health condition was, the worse their levels of anxiety, stress, depression, and sleep disorders were. It can be assumed that people suffering from chronic diseases were afraid of the war worsening their access to healthcare. Our findings are consistent with previous work suggesting that self-reported poor health is associated with higher DASS-21 scores and higher scores on the Impact of Event Scale—Revised (IES-R) [61].

Moreover, it has been observed that some mental disorders can influence certain patterns of reaction to a military crisis [61,62]. One study found that patients with depression were more aware of the horror of war than patients with schizophrenia, and they viewed the subjective risk of health consequences as being more probable [63]. Among other factors, dissatisfaction with one’s state of health was one of the most important factors causing mental disorders in a study conducted on a population of Ukrainian refugees after the outbreak of the RUW [64].

In the presented study, fear of war in a neighboring country and fear of its extension to the home country predisposed individuals to the development of mental disorders. Consistent with previous observations, experiencing such fears is a chronic source of stress and anxiety [65,66,67]. Moreover, research has found that people living with the specter of war and involved in helping refugees may feel overwhelmed and stressed by this situation [65,66,67]. Interesting findings have also been published by Kasierska et al., who examined Polish citizens three times: one month, two months, and six months after the RUW outbreak. Similarly to the present study, the authors also point to anxiety about the possibility of the area of military operations expanding and Poland being attacked as a risk factor for the occurrence of anxiety and depressive symptoms [68].

Riad et al. indicated that a potential risk factor for an escalated fear of war and the fear of its extension to the home country was a higher level of information-seeking behavior [5]. In the Polish study cited above, the risk factors for the occurrence of anxiety and depressive symptoms were found to be active monitoring of information about the RUW and frequent conversations about it [68].

In a real-life model, the coexistence of factors plays a key role in the development of mental disorders. As discussed in the Results section, when the depression, anxiety, stress, and insomnia scores were divided into two groups, severe and non-severe, logistic regression analysis was only possible for one variable: insomnia. Potential factors influencing this variable included five factors in the univariate logistic regression model (age, health status, depression, anxiety, and stress) but only three factors in the multivariate model (age, stress, and fear of armed conflict in Ukraine). This is consistent with the findings of Kasierska et al., who also indicated that, among other factors, sleep problems were associated with higher levels of anxiety and depression [68]. In a study conducted in 2022 among Ukrainian refugees, Boiko et al. reported a bi-directional relationship between anxiety and insomnia, with both symptoms being associated with subsequent psychiatric disorders [64].

In the present study, age was a predictor of the risk of insomnia. The older the age of respondents, the greater the risk of sleep disorders. Msaad et al. used multivariate binary logistic regression analysis to show that, among others, severe depression and a history of mental illness were found to be the strongest predictors of poor sleep quality. However, they proved that the youngest respondents were characterized by the highest rates of prevalence of both sleep and mood disorders, as well as dysfunctions during the day [47]. These findings are consistent with the results of a study assessing the mental state of Jordanians during the outbreak of the war in Gaza. Salem Gammoh et al. also indicated age as a predictor of sleep disorders among their study participants. The authors reported that severe insomnia was significantly associated with respondents “under 30 years of age” [69]. Similarly, young age was associated with greater concern about the RUW in a study conducted by Karakiewicz et al., the aim of which was to survey Polish citizens on the need to provide humanitarian aid to Ukrainian refugees [70].

Although age was found to be a risk factor in the present study, there is conflicting evidence in the literature. In a study of 178 Ukrainian combatants, age was not significantly associated with anxiety, depression, or insomnia [71]. This is one of the few studies conducted at the early stage of RUW among civilian and military veterans. Similarly, there was no association between age and insomnia in a study conducted in 2022 by Pavlova et al., who examined sleep disturbances among university students living in western Ukraine. Among them, 49% experienced insomnia symptoms and 27% presented symptoms of PTSD. Network analysis results showed that war-related PTSD significantly influenced the severity of their insomnia symptoms [72].

Numerous social factors—including unemployment, poor public transport and limited access to healthy food or good education, poverty, poor housing, discrimination, and human rights violations—have been identified as risk factors for mental health deterioration. All of them are strongly influenced by the government, its ideology, policies, and politics [73]. Successfully improving mental health management during any armed conflict depends on government conflict management measures. A good government approach requires appropriate funding policies, regular review of national disaster management programs, support for research programs, and capacity building at both the institutional level and the community level [74]. The impact of sociopolitical values and political ideologies on mental health requires further research.

Finally, it is important to emphasize that the consequences of war are neither linear nor equal for all affected societies [67,75]. Anjum et al. analyzed how helpers in host countries can provide significant support. They promoted certain principles, including recognizing strengths and solving problems, building good relationships between helpers, and recognizing different social–political contexts and histories. These researchers concluded that some people can quickly return to a “normal” life while others will suffer from numerous mental disorders, including fear, trauma, depression, PTSD, and alcohol dependence [67].

### 4.4. Practical Implications

The results of this study have both practical and social applications. In recent years, societies have faced numerous crises caused by wars, epidemics, natural disasters, and other catastrophes [76]. Any public health threat negatively impacts the mental health of the affected population. However, it is also pointed out that each crisis can be treated as both a challenge and an opportunity to build resilient public health systems [14]. Growing knowledge about the impact of such crises on mental health is a key basis for disaster intervention programming [77]. It should be emphasized that little research has been conducted so far on the indirect effects of the RUW, which makes the findings presented here particularly valuable.

Healthcare professionals play a key role in mental health recovery, especially since psychological support is irreplaceable in the early stages of a crisis. They can implement simple behavioral interventions in healthcare settings, which is very necessary because “simple” coping behaviors can effectively protect against symptoms of anxiety and depression in crisis situations [78]. Monitoring mental health symptoms during medical visits can be a helpful tool in distinguishing between transient reactions and the onset of a mental disorder.

Promoting healthy sleep habits, such as maintaining a regular sleep schedule, getting enough sleep, and practicing good sleep hygiene, is crucial [79,80]. It is also necessary to share knowledge about all aspects of a healthy lifestyle, including diet, which affects the quality of sleep [81]. Moreover, a relationship has been demonstrated between inappropriate eating behaviors and the occurrence of anxiety and depression [78,82]. Engaging in activities that evoke positive emotions can also be beneficial [77] and contribute to a better ability to cope with stress or traumatic experiences [83]. It has been proven that people who are physically active and have hobbies show only mild anxiety in crisis situations [78], but when they lose their daily routines and activities, they experience frustration and higher levels of stress [84].

Social support is important for maintaining mental health in a crisis situation [77]. The fact that good relationships with family and friends, spending time with loved ones, conducting conversations, and sharing rituals are crucial in coping with the crisis should be widely promoted [68,85]. Social support and resilience may be protective factors against mental health problems in people exposed to armed conflict, both during the conflict and many years after its end [86,87]. Such support and resilience may enhance perceived hope, which acts as a moderator influencing the positive effects of perceived threat and anxiety on posttraumatic growth (PTG). However, perceived hope was not found to significantly moderate the direct impact of perceived threats and anxiety on well-being in a study on the Czech adult population after the outbreak of the RUW [88]. These authors underlined that hope is critical in enabling people to perceive challenges instead of disasters and strategize their way through them. In other words, hope activates a transformative process, converting negative experiences into growth opportunities.

In the present study, the deterioration of mental health was observed especially among people with a poor self-assessment of their health, fear of the RUW, and fear of this war extending to Poland. Therefore, targeting mental health interventions and supports, as well as strengthening coping skills and resilience, is crucial. Efforts should focus on making mental health services visible and accessible to those in need.

Moreover, limiting access to news from the war front can help reduce the harmful impact of crises on individuals’ mental health. Important directions for future research include continuing to examine the negative effects of crises on mental health, using a variety of methods and measures, and focusing on the long-term effects as situations unfold or even after the crisis is resolved. Moreover, experimental testing of the effectiveness of appropriate mental health interventions remains a key research direction [89].

### 4.5. Limitations and Strengths

The strength of our analysis lies in the fact that it is one of the first assessments carried out just after the start of the RUW assessing the mental health of Polish citizens involved in providing various types of assistance to Ukrainian refugees. It should be noted that we also used standardized instruments that are recommended for use in crisis situations. The strength of the research tools used here is that they mostly comprise mandatory questions and do not allow the omission of any questions, unlike paper questionnaires.

The main shortcoming of this study is the non-random nature of the sample selection process due to the respondents’ limited access to the internet or limited interest in the subject. This study is also limited by the fact that the data were collected at one time point and in one country. When extrapolating the obtained results to the entire Polish population, the limited representativeness of the sample should be taken into account.

In the light of the presented findings, it is necessary for public health authorities to organize psychological assistance for civilians helping refugees in the event of an armed conflict. Our study has identified several high-risk groups. Therefore, future research should assess all factors that may affect the mental health of citizens during armed conflicts. The RUW is likely to continue, so it would be interesting to compare our data with similar data from other European countries.

## 5. Conclusions

Within the above-mentioned limitations of this study, the surveyed Polish citizens were found to be highly concerned about the RUW. Almost half of them (45.67%) manifested symptoms of anxiety, depression, and/or stress. Slightly less than two-thirds of them had sleep disturbances. Self-reported health status, fear of the Russian invasion of Ukraine, and fear of the war extending to Poland each had a decisive impact on the development of psychiatric symptoms among the study participants. After taking into account the co-occurrence of the analyzed factors, the strongest predictors of insomnia were found to be age, stress, and fear of the Russian invasion of Ukraine. This study confirms that mental health screening is necessary in a crisis situation.

Since the first weeks of the RUW, there has been much public discussion on how to optimize all kinds of resources— mental, social, and financial—in order to organize optimal assistance for both Ukrainian refugees and host countries. And that discussion is still relevant today.

## Figures and Tables

**Table 1:** Characteristics of Polish citizens who participated in this study *(n* = 311).

Group Size	Total	311
Sex	Female, *n* (%)	162 (52.09)
	Male, *n* (%)	149 (47.91)
Age	Overall mean ± (SD) *	33.45 ± (13.53)
Education	Higher, *n* (%) Secondary, *n* (%) Vocational, *n* (%)	200 (64.30) 106 (34.08) 5 (1.60)
Occupation	Employed, *n* (%)	174 (55.94)
	Retired, *n* (%)	6 (1.92)
	Student, *n* (%)	127 (40.83)
	Unemployed, *n* (%)	4 (1.28)
Place of residence	Village, *n* (%)	73 (23.47)
	Town of <50,000 inhabitants, *n* (%)	46 (14.79)
	Town of <100,000 inhabitants, *n* (%)	15 (4.82)
	City of 100,000–250,000 inhabitants, *n* (%)	17 (5.46)
	City of over 250,000 inhabitants, *n* (%)	160 (51.44)
Household	Living alone, *n* (%)	75 (24.11)
	Living with family (%)	236 (75.88)
Self-reported health status	Very good, *n* (%)	150 (48.22)
	Good, *n* (%)	133 (42.76)
	Not very good, *n* (%)	25 (8.03)
	Bad, *n* (%)	3 (0.96)
Comorbidities	Hypertension, *n* (%)	33 (10.61)
	Obesity, *n* (%)	35 (11.25)
	Major Depressive Disorder, *n* (%)	49 (15.75)
Use of stimulants	Alcohol use, *n* (%)	140 (45.01)
	Nicotine use, *n* (%)	58 (18.64)
	Narcotics use, *n* (%)	14 (4.50)

* SD = standard deviation.

**Table 2:** Assessment of the levels of anxiety, stress, mood disorders, and sleep disturbances among Poles after the outbreak of the armed conflict in Ukraine on 24 February 2022.

Level of Depression	Level of Anxiety	Level of Stress	Level of Sleep Disturbance
Normal [%]: 88.74	Normal [%]: 89.71	Normal [%]: 75.88	Not clinically significant [%]: 37.95
Mild [%]: 5.16	Mild [%]: 8.37	Mild [%]: 15.11	Subthreshold insomnia [%]: 51.12
Moderate [%]: 4.82	Moderate [%]: 1.92	Moderate [%]: 9.01	Clinical insomnia (moderately severe) [%]: 10.93
Severe [%]: 1.28	Severe [%]: 0.00	Severe [%]: 0.00	Clinical insomnia (severe) [%]: 0.00
Extremely severe [%]: 0.0	Extremely severe [%]: 0.00	Extremely severe [%]: 0.00	—
Overall [general mean ± SD *, Me ^#^]: 5.86 ± 3.96, 5	Overall [general mean ± SD, Me]:	Overall [general mean ± SD, Me]:	Overall [general mean ± SD, Me]:
	4.08 ± 3.07, 4	7.50 ± 3.56, 7	8.61 ± 5.38, 8

* SD = standard deviation; ^#^ Me = median.

**Table 3:** Polish citizens’ fears associated with the outbreak of the armed conflict in Ukraine following 24 February 2022.

Type of Fear	Number of Respondents (%)
Fear of the armed conflict extending to Poland or throughout Europe	190 (61.09)
Anxiety associated with deteriorating financial status	221 (71.06)
Fear about Russia’s invasion of Ukraine and the socioeconomic consequences of the armed conflict	227 (72.99)

**Table 4:** Outcomes of the analysis of psychiatric symptoms (depression, anxiety, stress, and sleep disturbance) among Polish citizens associated with the outbreak of the armed conflict in Ukraine following 24 February 2022, depending on the analyzed factors.

Variables	Level of Depression		Level of Anxiety		Level of Stress		Level of Insomnia	
	Median [Q_1_–Q_3_]	*p*-Value	Median [Q_1_–Q_3_]	*p*-Value	Median [Q_1_–Q_3_]	*p*-Value	Median [Q_1_–Q_3_]	*p*-Value
Gender		0.585		0.323		0.124		0.314
Female	5 [3–8]		4 [2–6]		7 [5–10]		8 [4–12]	
Male	6 [3–8]		3 [1–6]		7 [4–8]		6.5 [4–12]	
Education		0.255		0.029 ^#^		0.754		0.758
Vocational	8 [5–10]		6 [5–6]		7 [4–7]		7 [3–10]	
Secondary	6 [3–8]		4 [2–7]		8 [4–9]		7 [5–12]	
Higher	5 [3–8]		3 [1–6]		7 [5–10]		8 [4–13]	
Occupation		0.251		0.305		0.746		0.051
Employed	5 [3–8]		3 [2–6]		7 [6–9]		8 [4–13]	
Retired	5.5 [3–10]		4 [1–7]		8 [3–9]		10 [7–16]	
Working students	6 [3–8]		4 [2–7]		7 [5–10]		7 [4–11]	
Place of residence		0.267		0.091		0.803		0.178
Village	6 [4–8]		4 [2–7]		7 [6–10]		10 [7–12]	
Town of up to 50 thousand residents	6 [3–8]		4 [1–7]		8 [6–9]		7.5 [4–12]	
Town of up to 100 thousand residents	6 [4–8]		5 [4–7]		7 [6–10]		8 [6–10]	
City of up to 250 thousand residents	6 [2–6]		3 [2–6]		7 [4–8]		8 [4–12]	
City of more than 250 thousand residents	5 [3–8]		3 [1–5]		7 [5–10]		7 [4–12]	
Household		0.795		0.675		0.518		0.846
Living with family	5 [3–8]		3 [2–6]		7 [5.5–9]		8 [4–12]	
Living alone	5 [3–8]		4 [2–7]		7 [4–10]		8 [5–11]	
Self-reported health status		<0.001		<0.001		<0.001		<0.001
Very good	4 [2–6]	<0.001 *	3 [1–4]	<0.001 *	6 [4–8]	<0.001 *	6 [4–9]	<0.001 *
Good	6 [4–9]	<0.001 **	5 [2–7]	<0.001 **	8 [6–11]	<0.001 **	9 [6–13]	<0.001 **
Not very good	9 [8–13]	0.019 ***	7 [5–11]	0.041 ***	11 [8–13]	0.002 ***	12 [11–17]	0.036 ***
Bad	12 [2–21]		6 [5–8]		9 [5–14]		15 [11–16]	
Fear of the armed conflict in Ukraine		0.072		0.042 ^#^		<0.001		0.003
No	4 [2–8]		3 [1–5]		6 [4–8]	<0.001 ^$^	6 [4–11]	0.012 ^$^
Yes	6 [3–8]		4 [2–7]		8 [6–11]		9 [5–13]	
I have no opinion	4.5 [3–7.5]		3 [1.5–5]		7 [4.5–8]		7 [2.5–10.5]	
Fear of the armed conflict extending to Poland		0.024		0.092		<0.001		0.522
No	3.5 [2–7]	0.020 ^$^	3 [1–5]		6 [3–8]	<0.001 ^$^	8 [4–12]	
Yes	6 [3–8]		4 [2–7]		8 [6–10]		8 [5–12]	
I have no opinion	4 [3.5–8]		3 [2–5.5]		7 [5–8]		7 [3–11]	

^#^ The post hoc analysis did not denote any homogenous groups; ^$^ Dunn’s post hoc result for yes vs. no; * Dunn’s post hoc result for very good vs. good; ** Dunn’s post hoc result for very good vs. not very good; *** Dunn’s post hoc result for good vs. not very good.

**Table 5:** Results of the univariate and multivariable logistic regression model analyses regarding insomnia among Polish citizens associated with the outbreak of the armed conflict in Ukraine following 24 February 2022, depending on the analyzed factors.

Variables	Univariate Model		Multivariable Model	
	OR [95% CI]	*p*-Value	OR [95% CI]	*p*-Value
Age	1.023 [1.00, 1.05]	0.040	1.04 [1.01, 1.07]	0.004
Gender				
Female	1.0 (ref.)			
Male	0.77 [0.32, 1.82]	0.56		
Education				
Vocational	1.0 (ref.)			
Secondary	0.46 [0.05, 4.52]	0.508		
Higher	0.71 [0.08, 6.61]	0.767		
Occupation				
Retired	1.0 (ref.)			
Employed	0.74 [0.07, 6.87]	0.791		
Unemployed	2.0 [0.08, 51.59]	0.676		
Working students	0.46 [0.05, 4.39]	0.497		
Household				
Living with family	1.0 (ref.)			
Living alone	1.30 [0.63, 2.68]	0.485		
Place of residence				
Village	1.0 (ref.)			
Town of up to 50 thousand residents	1.66 [0.63, 2.68]	0.350		
Town of up to 100 thousand residents	0.56 [0.63, 2.68]	0.601		
City of up to 250 thousand residents	1.69 [0.63, 2.68]	0.479		
City of over 250 thousand residents	1.25 [0.63, 2.68]	0.605		
Self-reported health status				
Very good	1.0 (ref.)			
Good	4.53 [1.88, 10.92]	0.001		
Not very good	12.26 [3.99, 37.63]	<0.0001		
Bad	40.85 [3.29, 506.53]	0.004		
Fear of the armed conflict in Ukraine				
No	1.0 (ref.)		1.0 (ref.)	
Yes	1.82 [0.82, 4.01]	0.135	2.85 [1.02, 7.96]	0.046
I have no opinion	0.29 [0.03, 2.35]	0.245		
Fear of the armed conflict extending to Poland				
No	1.0 (ref.)			
Yes	0.83 [0.37, 1.88]	0.669		
I have no opinion	1.13 [0.31, 4.10]	0.849		
Depression				
	1.19 [1.10, 1.29]	<0.001		
Anxiety				
	1.23 [1.12, 1.37]	<0.001		
Stress				
	1.34 [1.20, 1.49]	<0.001	1.40 [1.24, 1.59]	<0.001

## Data Availability

The raw data supporting the conclusions of this article will be made available by the authors on request.

## Supplementary Materials

The following supporting information can be downloaded at https://www.mdpi.com/article/10.3390/healthcare12181848/s1: Supplementary Material S1: Study questionnaire.
