# Supplementary material for: The Prevalence and Correlates of Anxiety, Stress, Mood Disorders, and Sleep Disturbances in Poland after the Outbreak of the Russian–Ukrainian War 2022
Source: Healthcare (Basel). 2024 Sep 14;12(18):1848. doi: 10.3390/healthcare12181848 (PMC11431018; doi:10.3390/healthcare12181848)
Supplement: Supplementary file 1 [file healthcare-12-01848-s001.zip › healthcare-3194543-supplementary.pdf]

File S1 „Study questionnaire"

ASSESSMENT OF THE LEVEL OF ANXIETY, STRESS, MOOD DISORDERS AND SLEEP DISTURBANCES AMONG POLES AFTER THE OUTBREAK OF THE ARMED CONFLICT IN UKRAINE 24/02/2022.

Dear Sir/Madam,

Researchers of the Poznan University of Medical Sciences are conducting a study entitled "Assessment of the level of anxiety, stress, mood disorders and sleep disturbances among Poles after the outbreak of the armed conflict in Ukraine 24/02/2022".

We kindly ask you to answer the following questions regarding your mental state after the Russian Federation invaded Ukraine on February 24, 2022. The survey consists of 45 questions, which take about 10 minutes to solve. It aims to collect information necessary for a scientific analysis.

The survey is safe, free of charge, and anonymous. Personal data of respondents shall not be recorded. Data that could be used to identify the survey participants in the future shall not be used. Upon agreeing to participate in the survey, you will be asked to respond to questions in an online survey, which will be used to collect the required demographic information and your personal attitude and psychological condition after the outbreak of Russo-Ukrainian War (RUW).

Every respondent may approach the authors to ask for information regarding the survey questions and to obtain a report once the survey has been completed.

Consent to take part in the survey

I have read and understood the information pertaining to the survey. By responding to this questionnaire, I voluntarily consent to participate in the survey and I am aware that I can withdraw my consent to participate in the subsequent parts of the survey at any time without providing a reason.

Pursuant to the Polish law (Personal Data Protection Act of 10 May 2018), this survey shall be anonymous.

1. **Sex: ***
- Female
- Male

2. **Age: ***
- 18-24
- 25-40
- 41-60
- more than 60.

3. **Education: ***
- Primary
- Basic vocational
- High school

4. **Your place of residence: ***
- Village
- Town, up to 50 thousand residents
- Town, up to 100 thousand residents
- City, up to 250 thousand residents
- City, more than 250 thousand residents.

5. **In which province do you live ? ***
- Dolnośląskie (Lower Silesia)
- Kujawsko-Pomorskie (Kuyavian-Pomeranian)
- Lubelskie (Lubuskie)
- Lubuskie (Lublin province)
- Łódzkie (Łódź province)
- Małopolskie (Lesser Poland)
- Mazowieckie (Masovian)
- Opolskie (Opole province)
- Podkarpackie (Subcarpathian)
- Podlaskie (Podlaskie)
- Pomorskie (Pomeranian)
- Śląskie (Silesia)
- Świętokrzyskie (Świętokrzyskie)
- Warmińsko-Mazurskie (Warmian-Masurian)
- Wielkopolskie (Greater Poland)
- Zachodniopomorskie (West Pomerania)

6. **What is your professional activity status? ***
- Pupil/student
- Professionally active
- Unemployed
- Pensioner

7. **Do you live alone or with your family? ***
- Alone
- With my family

8. **How would you describe your current state of health? ***
- Excellent

- Very good
- Good
- Not so good
- Poor

9. Which chronic illness have you been diagnosed with (you may tick more than one option) *

  - Obesity
  - Diabetes
  - Dyslipidemia (high cholesterol and/or triglycerides)
  - Other metabolic diseases
  - Hypothyroidism (acquired thyroid atrophy)
  - Autoimmune thyroid disease (including Hashimoto's disease and Graves' disease)
  - Other endocrine diseases
  - Osteoarthritis
  - Rheumatoid arthritis (RA)
  - Other joint diseases
  - Arterial Hypertension (high blood pressure)
  - Ischemic heart disease (coronary artery disease)
  - Prior heart attack
  - Prior stroke
  - Other cardiovascular diseases
  - Chronic neurological diseases
  - Chronic diseases of the respiratory system
  - Chronic diseases of the digestive system
  - Chronic diseases of the genitourinary system
  - Cancer
  - Schizophrenia
  - Mood disorders (including depression)
  - Neurotic disorders (including anxiety disorders)
  - Personality disorder
  - Other mental illnesses
  - I have not been diagnosed with any chronic diseases

10. Do you use stimulants ? (you may tick more than one option) *

- No
- Yes, alcohol
- Yes, tobacco
- Yes, drugs

11. Have you recently felt a real threat of an armed conflict also in Poland or throughout Europe ? *

- Yes
- No
- I have no opinion on this

12. Have you recently experienced economic difficulties and uncertainty about the future? *

- o Yes
- o No
- o I have no opinion on this

13. Have you recently felt fear about Russia's invasion of Ukraine and the socio-economic consequences of the armed conflict? *

- o Yes
- o No
- o I have no opinion on this

Please read each sentence and tick the answer that indicates how much the statement applies to you during the last week. There are no right or wrong answers. Please don't take too long to answer.

The rating scale is as follows and applies to the last week:
Never - doesn't apply to me at all
Sometimes- Applies to me to some extent or once in a while
Often - Affects me to a great extent or for a long time
Always/Almost always - Affects me very much or most of the time.

It was very difficult for me to relax. *

- o Never
- o Sometimes
- o Often
- o Always/Almost always

My mouth felt dry. *

- o Never
- o Sometimes
- o Often
- o Always/Almost always

I couldn't experience any positive feelings. *

- o Never
- o Sometimes
- o Often
- o Always/Almost always

I have experienced breathing difficulties (e.g. breathing too fast, feeling short of breath without prior exercise. *

- o Never
- o Sometimes
- o Often
- o Always/Almost always

Was it hard for me to find the initiative to act? *

- o Never
- o Sometimes
- o Often

- Always/Almost always

I tended to overreact to situations. *

- Never
- Sometimes
- Often
- Always/Almost always

I felt tremors (e.g. in my hands). *

- Never
- Sometimes
- Often
- Always/Almost always

I used a lot of energy due to increased nervous excitability. *

- Never
- Sometimes
- Often
- Always/Almost always

I was afraid of a situation where I might panic and make a fool of myself. *

- Never
- Sometimes
- Often
- Always/Almost always

I felt that nothing would ever happen to me again. *

- Never
- Sometimes
- Often
- Always/Almost always

I felt nervous *

- Never
- Sometimes
- Often
- Always/Almost always

It was hard for me to relax. *

- Never
- Sometimes
- Often
- Always/Almost always

I felt down and sad. *

- Never
- Sometimes
- Often
- Always/Almost always

I couldn't stand anything interfering with what I was doing. *

- Never
- Sometimes
- Often
- Always/Almost always

I felt I was about to panic. *

- Never
- Sometimes
- Often
- Always/Almost always

I couldn't be happy about anything. *

- Never
- Sometimes
- Often
- Always/Almost always

I felt worthless. *

- Never
- Sometimes
- Often
- Always/Almost always

I felt I was rather oversensitive. *

- Never
- Sometimes
- Often
- Always/Almost always

I could feel my heart beating when I wasn't doing any physical effort. *

- Never
- Sometimes
- Often
- Always/Almost always

I felt scared for no reason. *

- Never
- Sometimes
- Often
- Always/Almost always

I felt that life had no meaning. *

- Never
- Sometimes
- Often
- Always/Almost always

Have the symptoms listed above worsened recently? *

- Yes
- No
- Hard to say
- Not applicable

Were the above-mentioned symptoms mainly due to concerns about the peace situation in Poland and throughout Europe? *

- Yes
- No
- Hard to say
- Not applicable

Please rate the current severity (during the last two weeks) of your sleep problems.

Difficulty falling asleep? *

- Lack
- Mild
- Moderate
- Heavy
- Very heavy

How satisfied/dissatisfied are you with your current sleep quality? (in room 0 to 4) *

Very satisfied

- 0
- 1
- 2
- 3
- 4

Very dissatisfied

How much do your sleep disorders affect your functioning during the day (e.g. fatigue during the day, functioning at work and performing daily duties, concentration, memory, mood, etc.)?

- No impact
- Slightly
- Moderately
- Significantly
- Very strong

How noticeable do you think your sleep problems are to others, considering their impact on your quality of life?

- Unnoticeable
- Barely
- To a certain extent
- Noticeable
- Very noticeable

How worried are you about your sleep disturbance?

- At all
- Slightly
- To a certain extent
- Significantly
- Very concerned
